# TET2 Protects against oxLDL-Induced HUVEC Dysfunction by Upregulating the CSE/H_2_S System

**DOI:** 10.3389/fphar.2017.00486

**Published:** 2017-07-26

**Authors:** Juan Peng, Zhi-Han Tang, Zhong Ren, Bei He, Yun Zeng, Lu-Shan Liu, Zuo Wang, Dang-Heng Wei, Xi-Long Zheng, Zhi-Sheng Jiang

**Affiliations:** ^1^Key Laboratory for Arteriosclerology of Hunan Province, Institute of Cardiovascular Disease, University of South China Hengyang, China; ^2^Department of Biochemistry and Molecular Biology, Health Sciences Center, The Libin Cardiovascular Institute of Alberta, University of Calgary, Calgary AB, Canada

**Keywords:** ten-eleven translocation-2, cystathionine-γ-lyase/hydrogen sulfide, endothelial dysfunction, DNA demethylation, oxidized low-density lipoprotein

## Abstract

Ten-eleven translocation-2 (TET2) protein is a DNA demethylase that regulates gene expression through DNA demethylation and also plays important roles in various diseases including atherosclerosis. Endothelial dysfunction represents an early key event in atherosclerotic disease. The cystathionine-γ-lyase (CSE)/hydrogen sulfide (H_2_S) is a key endogenous system with protective effects on endothelial functions. In this study, we examined how TET2 regulates oxidized low-density lipoprotein (oxLDL)-induced dysfunction of human umbilical vein endothelial cells (HUVECs) and determined the role of the CSE/H_2_S system. Treatment with oxLDL resulted in downregulation of both TET2 expression and CSE/H_2_S system in HUVECs. TET2 was found to have protective effects on oxLDL-induced HUVEC dysfunction, which was confirmed with TET2 overexpression plasmid or TET2 shRNA plasmid. Moreover, TET2 was found to upregulate the CSE/H_2_S system and inhibit NF-κB activation, leading to decreased expression of ICAM-1 and VCAM-1 and attenuated adhesion of THP-1 cells to oxLDL-activated HUVECs. The protective effect of TET2 was reduced by treatment with CSE siRNA. Further studies revealed that CSE promoter region contains a well-defined CpG island. We also showed that TET2 enhanced 5-hydroxymethylcytosine (5hmC) level and promoted DNA demethylation of CSE gene promoter, leading to an increase in CSE expression. In conclusion, TET2 has protective effects on oxLDL-induced HUVEC dysfunction, likely through upregulating the CSE/H_2_S system by DNA demethylation of CSE gene promoter. TET2 may become a novel therapeutic target for endothelial dysfunction-associated vascular diseases.

## Introduction

Atherosclerosis is a common pathological etiology of various cardiovascular diseases (Glass and Witztum, 2001). The pathogenesis of atherosclerosis is quite complex with various theories and hypotheses. It is well accepted that vascular endothelial dysfunction is the initial event in atherosclerosis (Davignon and Ganz, 2004; Landmesser et al., 2004). Oxidized low-density lipoprotein (oxLDL) is an important pathogenic factor associated with endothelial dysfunction in atherosclerosis (Mitra et al., 2011). OxLDL stimulates endothelial cells to secrete a variety of adhesion molecules and chemotactic factors and promotes the adhesion of monocytes to endothelial cells, leading to the migration to the intima (Devaraj and Jialal, 1996; Itabe, 2009). The monocytes in the intima differentiate into macrophages, which phagocytize excess lipids, finally leading to the formation of foam cells (Steinberg and Witztum, 2010; Ley et al., 2011).

Endogenous hydrogen sulfide (H_2_S) is the third gaseous molecule following nitric oxide (NO) and carbon monoxide (CO). It has been widely involved in various physiological and pathological processes (Huang and Moore, 2015). In the cardiovascular system, H_2_S is physiologically generated by cystathionine-γ-lyase (CSE) (Zhao et al., 2001; Ishii et al., 2004). It has been found that the defects of endogenous CSE/H_2_S system promote the development of atherosclerosis (Wang et al., 2009; Mani et al., 2013), whereas up-regulation of endogenous CSE/H_2_S pathway suppresses atherosclerosis (Cheung et al., 2014). The protection of H_2_S on endothelial functions is the main mechanism underlying H_2_S inhibition of atherosclerosis (Altaany et al., 2014). To date, the CSE/H_2_S system has already become an important regulator for atherosclerosis therapy (Mani et al., 2014; Xu et al., 2014). Exploring the mechanisms regulating the CSE/H_2_S pathway and search for potential targets to regulate this system are important for protecting the function of vascular endothelial cells and inhibiting the progression of atherosclerosis.

More recently, epigenetics has been increasingly appreciated to play a key role in atherosclerosis through altering gene expression and cell functions (Byrne et al., 2014; Loscalzo and Handy, 2014; Bauer and Martin, 2017). DNA methylation, one of the epigenetic modifications, predominantly occurs in CpG dinucleotides to induce chromatin structure changes which are often associated with gene repression (Minarovits et al., 2016). Ten-eleven translocation-2 (TET2) protein is a DNA demethylase that oxidizes 5-methylcytosine (5mC) to generate 5-hydroxymethylcytosine (5hmC) and promote DNA demethylation and activation of gene expression (Veron and Peters, 2011; Liu et al., 2013). It was reported that the expression of TET2 and 5-hmC in human atherosclerotic plaques is significantly lower than that in normal blood vessels (Liu et al., 2013). TET2 levels are inversely correlated with the severity of atherosclerosis (Liu et al., 2013). Our previous studies have found that TET2 inhibits atherosclerosis in ApoE knockout mice (Peng et al., 2016). We also found that TET2 is involved in regulation of endothelial cell functions under low shear stress (Yang et al., 2016). However, the relationship between TET2 and the CSE/H_2_S system and its role in endothelial dysfunction remain unclear.

Here, we first examined the intracellular TET2 expression and the change of CSE/H_2_S system in the oxLDL-treated human umbilical vascular endothelial cells (HUVECs). Then, we further determined whether TET2 regulates oxLDL-induced dysfunction of HUVECs via the CSE/H_2_S system, and investigated the underlying mechanism in this progress in term of DNA demethylation.

## Materials and Methods

### Cell Culture and Treatment

Human umbilical vascular endothelial cells were purchased from the China Center for Type Culture Collection and cultured as previously described (Yang et al., 2016). Briefly, HUVECs were cultured at 37°C with 5% CO_2_ in Dulbecco’s Modified Eagle’s medium (DMEM, GIBCO) containing 10% FBS. Cells were treated with different concentrations of oxLDL and also treated for different time periods.

### Cell Transfection

Human umbilical vascular endothelial cells (4 × 10^5^ cells per well) were seeded in a six-well plate and then transfected with the TET2 plasmid for overexpression (OriGene Technologies Inc.) or TET2 shRNA (OriGene Technologies Inc.) using Lipofectamine^®^2000 (Invitrogen) in accordance with the manufacturer’s instruction. After 6 h, the transfection mixture was replaced with fresh growth medium. Co-transfection with TET2 overexpression plasmid and CSE siRNA (Guangzhou RiboBio Co., Ltd.) in HUVECs was carried out according to siRNA plasmid co-transfection protocol with Lipofectamine^®^2000. Subsequent experiments with transfected cells were performed after transfection for 24 h.

### Detection of H_2_S Contents in Cells

Hydrogen sulfide generation in cultured HUVECs was examined as previously described (Xie et al., 2013). Briefly, filtration membranes were pretreated by zinc acetate solution and pasted on the inside of the plate lid. Cells were then cultured for 8 h. H_2_S released from HUVECs was trapped by zinc acetate in the filtration membrane to generate ZnS deposition. Then the ZnS deposition was measured by methylene blue assay. The absorbance of the resulting solution was measured with a spectrometer at a wavelength of 655 nm. The H_2_S concentration in the solution was calculated according to the calibration curve of the standard H_2_S solution.

To image the intracellular H_2_S levels, a highly selective and sensitive H_2_S probe-N3 obtained from Dr. J. L. Wang (Hunan University, China) was used. H_2_S Probe-N3 was added in the medium as the final concentration of 20 μmol/L. After 30 min incubation, cells were washed with PBS three times to remove the excess probe. Fluorescence images were taken with a fluorescence microscope (NikenE600, Tokyo).

### Adhesion Assay

Upon completion of indicated transfection, HUVECs were incubated with 75 μg/ml oxLDL for 24 h. Then, 1 × 10^5^ THP-1 cells were seeded onto confluent HUVECs, followed by 30 min incubation. Non-adherent THP-1 cells were removed by washing with PBS. The number of adhered THP-1 cells to HUVECs was observed and counted with an Olympus optical microscope system. The results were expressed as the mean number of cells per optical field at Scale bar = 50 μm.

### Immunostaining

Cells were fixed with 4% paraformaldehyde for 10 min, washed thee with PBS, and treated with 0.1% Triton X-100 for 10 min. Then cells were blocked in 10% normal goat serum for 30 min. The cell samples were incubated with primary antibodies for NF-κB p65 (1:200, Proteintech), 5-hmC (1:200, Epigentek) at 4°C overnight. After washed with PBS, cells were incubated with Cy3-conjugated affinipure goat anti-Rabbit IgG (1:100, Proteintech) or anti-Mouse IgG (1:100, Proteintech). The nuclei were counterstained with 4′,6-Diamidino-2-Phenylindole (DAPI). Immunofluorescence images were obtained using a Nikon E600 fluorescence microscope.

### Real-Time PCR

Total RNA was isolated using Trizol reagent (Shanghai Pu Fei Biotechnology Co., Ltd.) following the manufacturer’s instructions. The cDNA was prepared with the First-Strand Synthesis System (Promega), and then real-time PCR was carried out with the SYBR green PCR Master Mix (Applied Biosystems). Quantitative evaluation was analyzed using the Ct method. GAPDH expression was used as the internal control. The primer sequences were listed in Supplementary Table S1.

### Western Blotting Analysis

Cells were washed twice with chilled PBS and lysed with radioimmunoprecipitation assay buffer (RIPA buffer) for protein extraction as previously described. The primary antibodies used include GAPDH (1:1000, Hangzhou Goodhere Biotechnology Co., Ltd.), TET2 (1:1000, Proteintech), CSE (1:1000, Proteintech), ICAM-1 (1:1000, Proteintech), VCAM-1 (1:500, Santa Cruz), IκBα (1:1000, Proteintech), NF-κB p65 (1:1000, Proteintech), and Histone H3 (1:5000, Abcam). The chemiluminescence immunoblotting detection system (Shanghai Tanon, China) was used to analyze immunoreactive protein bands.

### DNA Methylation Analysis

DNA methylation analysis was carried out as previously described. Briefly, Genomic DNA Clean & Concentrator^TM^ Kit (D4011, Zymo Research) was used to extract genomic DNA from HUVECs. EZ DNA Methylation-Direct^TM^ Kit (D5020, Zymo Research) was applied to complete bisulfite conversion of genomic DNA in accordance with the manufacturer’s protocols. Bisulfite sequencing primers were designed by MethPrimer software. Upon ligation, the purified bisulfite PCR product of samples was cloned into the pBLUE-T vector system [ZC204, ComingTech InnoBIO (Beijing) Co., Ltd.]. After bacterial transformation, at least five bacterial colonies on the dish plates were selected and sent for direct sequencing in GenomeLab^TM^ GeXP Genetic Analysis System (Beckman Coulter). The sequencing data were analyzed by the BiQ Analyzer software. The primer sequences were for BSP listed in Supplementary Table S1.

### Statistical Analysis

Data were presented as mean ± SD. Statistical analyses were performed with the GraphPad Prism 5.0 Software. Differences between groups were analyzed with one-way analysis of variance (ANOVA). Differences were considered statistically significant when *p* < 0.05.

## Results

### OxLDL Downregulates TET2 Expression and the CSE/H_2_S System in HUVECs

**FIGURE 1:**
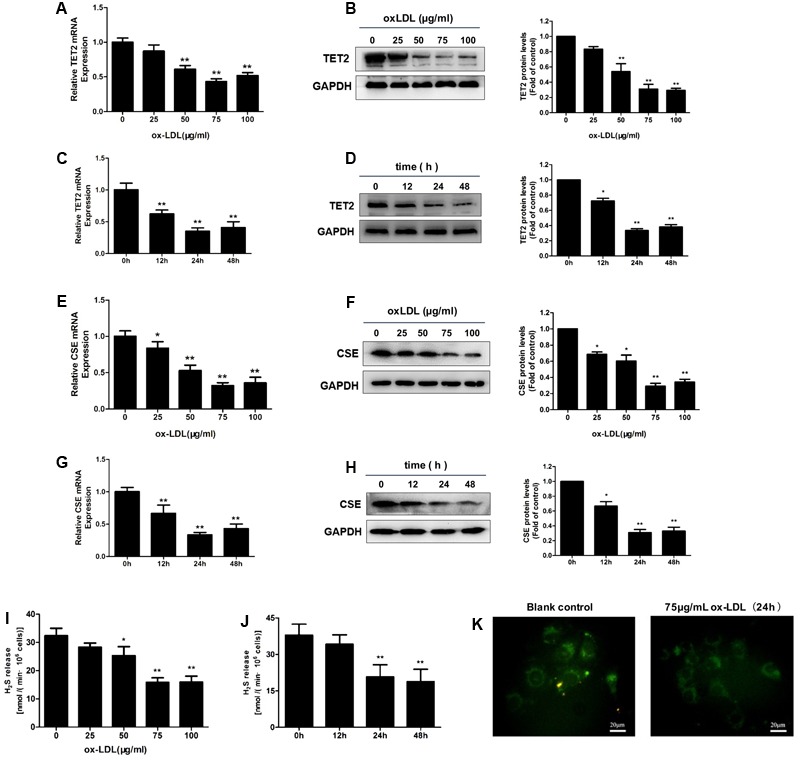
Effect of oxLDL on TET2 expression and the CSE/H_2_S system in HUVECs. **(A–D)** OxLDL reduced TET2 mRNA and protein expression in HUVECs at the concentration-dependent fashion (0, 25, 50, 75, and 100 μg/ml). 75 μg/ml oxLDL decreased TET2 mRNA and protein expression in HUVECs at the time-dependent manner (0, 12, 24, and 48 h). ^∗^*P* < 0.05, ^∗∗^*P* < 0.01 vs. 0 μg/ml oxLDL group **(A,B)** or vs. 0 h group **(C,D)**. **(E–H)** OxLDL reduced CSE mRNA and protein expression in HUVECs at the concentration-dependent fashion (0, 25, 50, 75, and 100 μg/ml) and 75 μg/ml oxLDL decreased CSE mRNA and protein expression in HUVECs at the time-dependent fashion (0, 12, 24, and 48 h). ^∗^*P* < 0.05, ^∗∗^*P* < 0.01 vs. 0 μg/ml oxLDL group **(E,F)** or vs. 0 h group **(G,H)**. **(I,J)** The effects of increasing concentrations of oxLDL on the H_2_S production rates in cells and the effects of 75 μg/ml oxLDL on H_2_S production rates in cells over time. ^∗^*P* < 0.05, ^∗∗^*P* < 0.01 vs. 0 μg/ml oxLDL group **(I)** or vs. 0 h group **(J)**. **(K)** Detection of intracellular H_2_S levels in HUVECs with or without 75 μg/ml oxLDL treatment for 24 h using H_2_S-specific fluorescent probes. Representative fluorescent images were taken using a fluorescent microscope. Scale bar = 20 μm. All results are expressed as the mean ± SD of three independent experiments.

Human umbilical vascular endothelial cells were incubated with different concentrations of oxLDL and treated for different time periods. OxLDL treatment of HUVECs resulted in an obvious decrease in TET2 mRNA and protein expression. The decrease in response to oxLDL treatment was in both concentration- and time-dependent manners (**Figures 1A–D**). The levels of CSE mRNA and protein also were downregulated in a concentration- and time-dependent fashion by oxLDL in HUVECs (**Figures 1E–H**). In line with the change of CSE expression, H_2_S production rate and level were significantly reduced in HUVECs treated with oxLDL as shown in **Figures 1I–K**. Therefore, the above results revealed that the downregulation of the CSE/H_2_S system in oxLDL-stimulated HUVECs was consistent with the alteration of TET2 expression.

Since treatment with 75 μg/ml oxLDL for 24 h resulted in a consistent and predictable response in terms of TET2 expression and the CSE/H_2_S system change in HUVECs, the subsequent experiments were performed with this concentration of oxLDL and treatment time.

### TET2 Improves oxLDL-Induced Dysfunction of HUVECs

**FIGURE 2:**
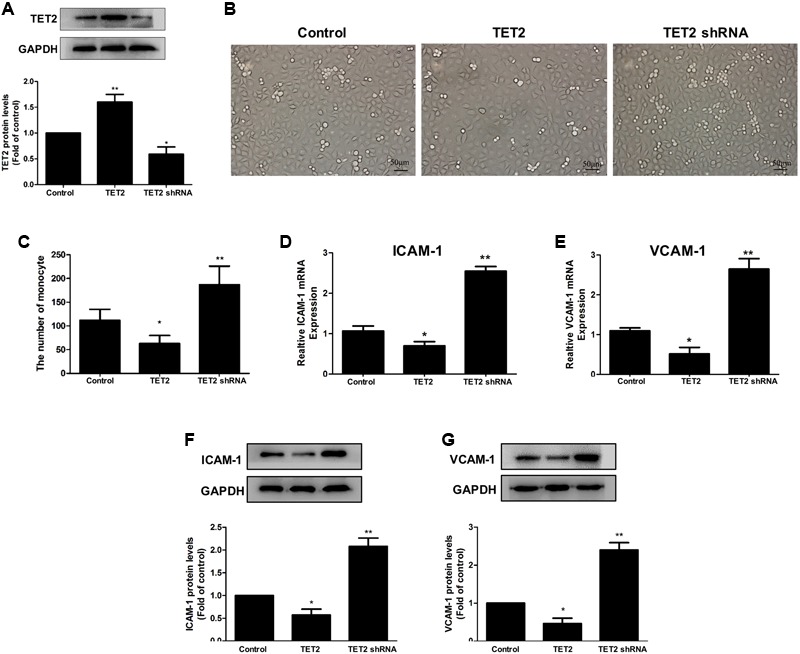
Effects of TET2 on the oxLDL-induced dysfunction of HUVECs. **(A)** The effects of TET2 overexpression and shRNA plasmids on TET2 protein expression in HUVECs. **(B,C)** HUVECs were transduced with TET2 overexpression plasmid or TET2 shRNA plasmid, then treated with 75 μg/ml oxLDL for 24 h. THP-1 cells were seeded onto HUVECs and co-cultured for 30 min. After washing the non-adherent cells, adherent cells were detected and counted under a light microscope. Representative light microscopic pictures of THP-1 cell adhesion to oxLDL-activated HUVECs **(B)** and quantitative analysis of adhesion results **(C)**. Data are the mean ± SEM of results from at least three independent experiments, each performed in duplicate. ^∗^*P* < 0.05, ^∗∗^*P* < 0.01 vs. control (treated with oxLDL alone) group. **(D–G)** The mRNA and protein levels of ICAM-1 and VCAM-1 in oxLDL-treated HUVECs with TET2 overexpression or TET2 silencing were determined by real-time PCR and western blot analyses. All results are expressed as the mean ± SD of three independent experiments, ^∗^*P* < 0.05, ^∗∗^*P* < 0.01 vs. control (treated with oxLDL alone) group. All results are expressed as the mean ± SD of three independent experiments, ^∗^*P* < 0.05, ^∗∗^*P* < 0.01 vs. control group.

To explore the role of TET2 in oxLDL-induced dysfunction of HUVECs, we chose oxLDL-treated HUVECs as a cell model and transduced the cells with TET2 overexpression plasmid or TET2 shRNA plasmid for TET2 overexpression or silencing, respectively (**Figure 2A**). Then, we first investigated the effect of TET2 on the adhesion of THP-1 cells to oxLDL-activated HUVECs, which indicates the function of HUVECs. As shown in **Figures 2B,C**, the adhesion of THP-1 cells to oxLDL-activated HUVECs was attenuated by TET2 overexpression. However, TET2 silencing markedly increased adhesion of monocytes to HUVECs treated with oxLDL.

ICAM-1 and VCAM-1 are considered as the key adhesion molecules that are induced by oxLDL in HUVECs, which then promote the adhesion of monocytes to HUVECs (Erl et al., 1998). The results of real-time PCR and western blot analyses demonstrated that ICAM-1 and VCAM-1 mRNA and protein expressions were decreased in oxLDL-treated HUVECs with TET2 overexpression and increased in oxLDL-treated HUVECs with TET2 silencing compared with those in cells treated with oxLDL alone (**Figures 2D–G**). These data indicate that TET2 results in an improvement of endothelial dysfunction induced by oxLDL.

### TET2 Upregulates the CSE/H_2_S System in oxLDL-Treated HUVECs

**FIGURE 3:**
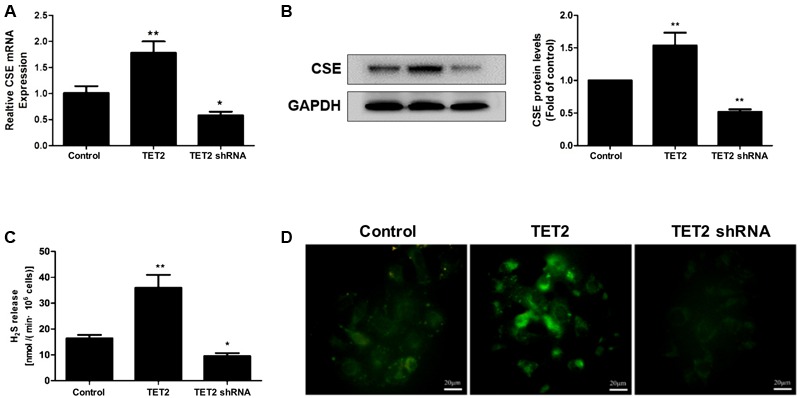
Effects of TET2 on the CSE/H_2_S system in oxLDL-induced dysfunction of HUVECs. HUVECs were transfected with or without TET2 overexpression plasmid or TET2 shRNA plasmid in the presence of oxLDL for 24 h. The expression of CSE mRNA **(A)** and protein **(B)** was examined via real-time PCR and western blot analyses in cells. **(C)** The H_2_S production rates in each group of cells were determined as described in “Materials and Methods” section. **(D)** Representative fluorescent images of intracellular H_2_S levels in each group of cells using H_2_S-specific fluorescent probes. Scale bar = 20 μm. All results are expressed as the mean ± SD of three independent experiment. ^∗^*P* < 0.05, ^∗∗^*P* < 0.01 vs. control group.

Next, the experiments were carried out to investigate the impact of TET2 on the CSE/H_2_S system in oxLDL-treated HUVECs. TET2 overexpression resulted in a remarked increase in the mRNA and protein mass of CSE in HUVECs (**Figures 3A,B**) along with an enhanced H_2_S production rate and an increased intracellular H_2_S level (**Figures 3C,D**). In line with these findings, silencing of TET2 led to the suppression of CSE mRNA and protein expression, resulting in low H_2_S production rate and intracellular H_2_S level in oxLDL-treated HUVECs (**Figure 3**).

### TET2 Inhibits NF-κB Activation in oxLDL-Treated HUVECs

**FIGURE 4:**
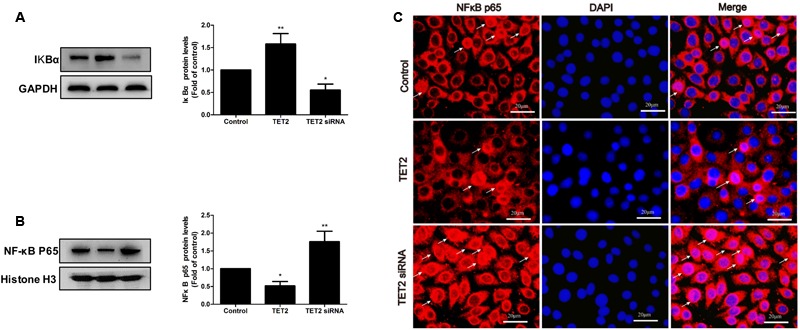
Effects of TET2 on NF-κB activation in oxLDL-induced dysfunction of HUVECs. HUVECs were transfected with or without TET2 overexpression plasmid or TET2 shRNA plasmid in the presence of oxLDL for 24 h. The levels of IkBα protein **(A)** and nuclear NF-κB p65 protein **(B)**, respectively, were evaluated by western blot analysis in each group of cells. All results are expressed as the mean ± SD of three independent experiment. ^∗^*P* < 0.05, ^∗∗^*P* < 0.01 vs. control group. **(C)** Distribution of NF-κB p65 protein expression was detected by immunostaining in each group of cells. NF-κB p65 positive staining is red. NF-κB p65 accumulation in the nuclei of the cells shows pink. DAPI staining is blue. Scale bar = 20 μm.

NF-κB, a major target molecule at the downstream of H_2_S, is the key regulator of ICAM-1 and VCAM-1 expression. So, we examined the modulation of NF-κB activation by TET2 overexpression plasmid and TET2 shRNA plasmid in oxLDL-treated HUVECs. Transfection of TET2 overexpression plasmid to cells led to an inhibition of IkBα degradation and NF-κB p65 nuclear translocation, whereas transfection with TET2 shRNA plasmid significantly promoted IkBα degradation and NF-κB p65 nuclear translocation in oxLDL-treated HUVECs (**Figure 4**). Thus, these data point to an inhibitory role of TET2 in NF-κB activation in oxLDL-treated HUVECs.

### The CSE/H_2_S System Mediates the Improvement Effect of TET2 on oxLDL-Induced Dysfunction of HUVECs

**FIGURE 5:**
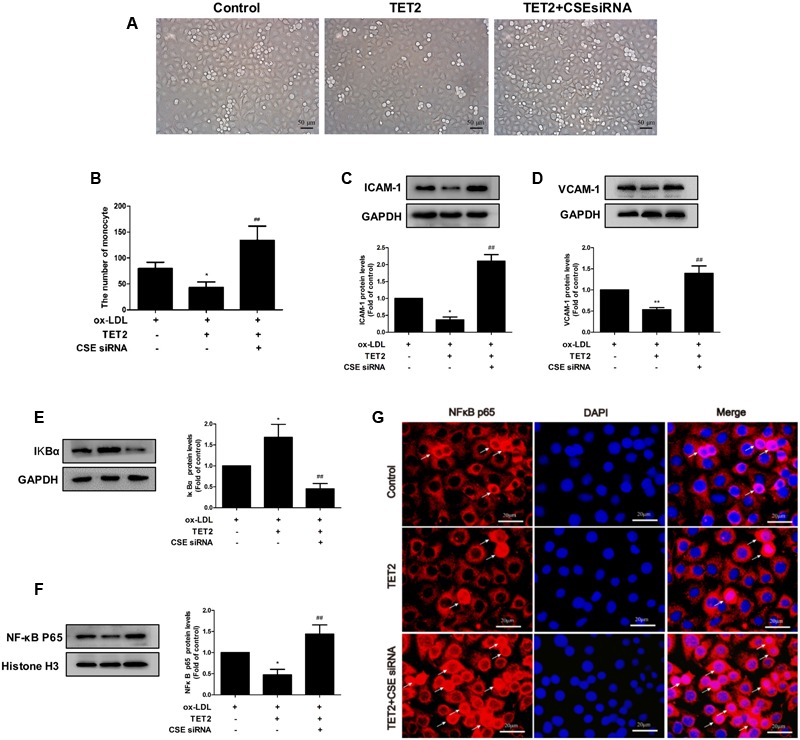
Effects of the CSE/H_2_S system on the TET2-induced improvement of oxLDL-treated dysfunction of HUVECs. HUVECs were transfected with or without TET2 overexpression plasmid or TET2 overexpression plasmid + CSE siRNA in the presence of oxLDL for 24 h. **(A,B)** Representative microscopic images of the adhesion of THP-1 cells to HUVECs **(A)** and quantitative analysis of adhesion results. Data are the mean ± SEM of results from at least three independent experiments. **(B).** The ICAM-1 **(C)** and VCAM-1 **(D)** proteins were evaluated by western blot analysis in each group of cells. The levels of IkBα protein **(E)** and nuclear NF-κB p65 protein **(F)**, respectively, were examined by western blot analysis in each group of cells. **(G)** Distribution of NF-κB p65 protein expression was detected by immunostaining in each group of cells. NF-κB p65 positive staining is red. NF-κB p65 accumulation in the nuclei of the cells shows pink. DAPI staining is blue. Scale bar = 20 μm. All results are expressed as the mean ± SD of three independent experiments. ^∗^*P* < 0.05, ^∗∗^*P* < 0.01 vs. oxLDL-treated alone group. ^##^*P* < 0.01 vs. TET2 overexpression plasmid-treated group.

Subsequently, we determined whether the CSE/H_2_S system mediates the improvement effect of TET2 on oxLDL-induced dysfunction of HUVECs. To do so, we interfered the CSE/H_2_S system using chemically synthesized CSE siRNA in oxLDL-treated HUVECs with TET2 overexpression. As expected, CSE siRNA suppressed CSE protein expression (**Supplementary Figure S1A**) and reduced H_2_S production rate (**Supplementary Figure S1B**) and intracellular H_2_S level in cells (**Supplementary Figure S1C**). As shown in **Figures 5A–D**, CSE siRNA ameliorated the suppression effect of TET2 overexpression on the adhesion of THP-1 cells to oxLDL-activated HUVECs and the levels of ICAM-1 and VCAM-1 protein in cells. In addition, the inhibition effect of TET2 overexpression on IkBα degradation and NF-κBp65 nuclear translocation was blocked by CSE siRNA in oxLDL-treated HUVECs (**Figures 5E–G**). Collectively, these data demonstrated that the CSE/H_2_S system mediates the improvement effect of TET2 on the oxLDL-induced dysfunction of HUVECs.

### TET2 Induces Demethylation of CSE Promoter in oxLDL-Treated HUVECs

**FIGURE 6:**
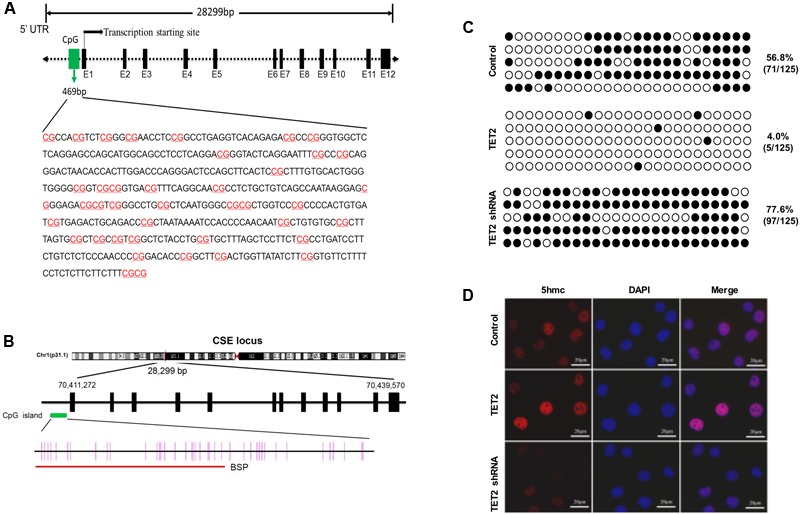
Effects of TET2 on the methylation level of the CSE promoter in oxLDL-treated HUVECs. **(A)** The features of CpG Island in human CSE gene promoter region was analyzed using the UCSC Human Genome Browser (http://genome.ucsc.edu/). **(B)** A schematic diagram of the CpG dinucleotides within the CSE promoter. The nucleotide number is relative to the transcription start site of CSE. The red line indicates the region that was tested with BSP. **(C)** HUVECs were transfected with or without TET2 overexpression plasmid or TET2 shRNA plasmid in the presence of oxLDL for 24 h. The methylation levels of the CSE promoters in each group of cells were determined by BSP method. Each row represents an individual clone sequenced; black and white circles represent methylated and unmethylated CpGs sites, respectively. The number of methylated CpGs divided by the whole CpG sites examined is given as a percentage of methylation. **(D)** Immunostaining for 5hmC in each group of cells. 5hmC is red, DAPI staining is blue, *n* = 4. Scale bar = 20 μm.

To elucidate the potential mechanism underlying TET2 regulation of the CSE/H_2_S system in oxLDL-treated HUVECs, we performed studies to examine the impact of TET2 on methylation status of the CSE promoter. Bioinformatics analysis showed that the CSE promoter contained a CpG island which extended across 469 bp (**Figure 6A**), harboring a CG content of 61.6% with an observed-to-expected CpG ratio of 0.91, suggesting a well-defined CGI compared with the CpG island definition standard (**Table 1**). These data of bioinformatics analyses suggest that the CSE promoter has the high probability to be modified by DNA methylation.

**Table 1:** CpG island of cystathionine-γ-lyase (CSE) promoter region contrasted with the standard CpG island.

Criteria	CGI definition Standard	CSE CGI
DNA stretch (bp)	≥200	469
GC content (%)	>50%	61.6
Obs/Exp radio	>0.6	0.91

*Obs/Exp radio, observed and expected ratio; CGI, CpG island.*

Then, the methylation level of the CSE promoters in oxLDL-treated HUVECs with TET2 overexpression plasmid or TET2 shRNA plasmid was determined by BSP method. The region of the CpG dinucleotides within the CSE promoter tested with BSP was indicated by red line in **Figure 6B**. As shown in **Figure 6C**, the methylation level of the CSE promoter was 56.8% in HUVECs treated with oxLDL alone, but it was decreased up to 4% in oxLDL-treated HUVECs with TET2 overexpression and increased in oxLDL-treated HUVECs with the TET2 knockdown. 5hmC represents an intermediate product in the TET2 active DNA demethylation process. We also evaluated the impact of TET2 on the 5hmC level in these cells by immunostaining. As expected, the level of 5hmC was enhanced by TET2 overexpression plasmid and decreased by TET2 shRNA plasmid (**Figure 6D**).

Taken together, these results suggest that TET2 could upregulate CSE expression via DNA demethylation, resulting in an increased production of H_2_S in oxLDL-treated HUVECs.

## Discussion

In the present work, we confirmed that TET2 expression and the CSE/H_2_S system were downregulated by oxLDL in HUVECs. Furthermore, we found that TET2 can upregulate the CSE/H_2_S system and inhibit NF-κB activation, thus decrease the expressions of ICAM-1 and VCAM-1 and attenuate the adhesion of THP-1 cells to oxLDL-activated HUVECs. Notably, we demonstrated that TET2 increases CSE expression by promoting the demethylation of the CSE promoter in oxLDL-treated HUVECs. Taken together, our results showed a novel epigenetic pathway, by which TET2 upregulates the CSE/H_2_S system, leading to the protection of endothelial functions.

Oxidized low-density lipoprotein, an independent risk factor for atherosclerosis (Gomez et al., 2014), plays a casual role in endothelial dysfunction (Mitra et al., 2011). Our data showed that TET2 mRNA and protein expressions are reduced by oxLDL in concentration- and time-dependent fashion in HUVECs, which is consistent with the results from oxLDL-treated macrophages as we previously reported (Li G. et al., 2015). It was reported that oxLDL downregulates the CSE/H_2_S system in THP-1 and Raw264.7 macrophages (Zhao et al., 2011; Wang et al., 2013). We also demonstrated that oxLDL decreases the CSE expression and H_2_S production rate and level in concentration- and time-dependent manners in HUVECs. These data firstly confirm that TET2 level is positively correlated with CSE expression and H_2_S level in oxLDL-treated HUVECs.

The high expression of adhesion molecules, such as ICAM-1 and VCAM-1, leading to an abnormal increase in adhesion ability onto endothelial cell surface, is an important feature of endothelial dysfunction (Szmitko et al., 2003). It was confirmed that ICAM-1 is expressed in human atherosclerotic plaques. OxLDL can increase the expression of ICAM-1 on the endothelial cell surface (Mulvihill et al., 2002; Pina-Canseco Mdel et al., 2012; Zhao et al., 2016). VCAM-1 is another important adhesion molecule in vascular endothelial cell surface, which can promote the adhesion of monocytes and T lymphocytes to endothelial cells (Hope and Meredith, 2003). In this study, we found that TET2 overexpression reduced the expression of ICAM-1 and VCAM-1, and inhibited the adhesion THP-1 cells to oxLDL-activated HUVECs. However, TET2 silencing had opposite effects. Recent studies have shown that TET2 affects atherosclerosis progression. Fuster et al. (2017) found that TET2 knockout in macrophages aggravates inflammation and accelerates atherosclerosis in LDLR-/- mice. Our group previously reported that TET2 improves low shear stress induced-endothelial cell dysfunction (Yang et al., 2016), and inhibits atherosclerosis via upregulating autophagy activity and downregulating the expression of inflammation factors in ApoE-/- mice (Peng et al., 2016). Given that the oxLDL-induced endothelial dysfunction plays a critical role in atherosclerosis, our finding that TET2 can improve the endothelial dysfunction induced by oxLDL will further support an inhibitive effect of TET2 on atherosclerosis.

It is well known that the CSE/H_2_S system has a protective effect on endothelial cell functions (Pan et al., 2011; Guan et al., 2013; Shen et al., 2013; Wen et al., 2013; Zong et al., 2015; Kanagy et al., 2017). Pan et al. (2011) and Guan et al. (2013) have found that H_2_S decreases the ICAM-1 and VCAM-1 expressions in endothelial cells induced by TNF-α or high glucose and improves the endothelial dysfunction. We examined whether TET2 improvement of oxLDL-induced endothelial dysfunction was linked to the CSE/H_2_S system. Our results have illustrated that TET2 overexpression results in an enhanced expression of CSE mRNA and protein with an increase in H_2_S production rate and H_2_S levels in oxLDL-treated HUVECs. To our knowledge, this is the first report to demonstrate that TET2 upregulates the CSE/H_2_S system in HUVECs. Importantly, we have shown that the inhibitory effects of TET2 on the expressions of ICAM-1 and VCAM-1 and the adhesion of THP-1 cells to HUVECs were reversed when the CSE/H_2_S pathway was interrupted by CSE siRNA in oxLDL-treated HUVECs, suggesting a role for the CSE/H_2_S system in TET2 protection of endothelial functions.

Furthermore, we have shown that TET2 overexpression inhibited NF-κB activation in oxLDL-treated HUVECs, whereas the TET2 silencing had the opposing effects. It is known that NF-κB directly binds to the promoters of ICAM-1 and VCAM-1 genes and stimulates their gene expression (Iademarco et al., 1992; Marui et al., 1993; Bauer and Martin, 2017). H_2_S is known to inhibit the activation of NF-κB in endothelial cells or macrophages in response to treatment with various stimuli (Oh et al., 2006; Wang et al., 2009; Pan et al., 2011; Guan et al., 2013). Therefore, it is conceivable that TET2 inhibits NF-κB activation through upregulating the CSE/H_2_S system, leading to a decrease in the expression of ICAM-1 and VCAM-1 in HUVECs. Indeed, the interference of the CSE/H_2_S pathway with CSE siRNA ameliorated the inhibitory effect of TET2 overexpression on NF-κB activation as shown in our results. In sum, our findings have suggested that the anti-atherosclerotic effect of TET2 may be mediated by the CSE/H_2_S system, but more *in vivo* studies will be required to establish the role of the TET2/CSE/H_2_S pathway in atherosclerosis.

DNA methylation and demethylation are two forms of epigenetic modifications. When located in a gene promoter, DNA methylation usually represses gene transcription, and DNA demethylation induces activation of gene transcription (Mueller and von Deimling, 2009). TET2 effects are mediated by site-specific DNA demethylation through oxidizing 5mC into 5hmC, which is associated with gene transactivation in mammalian cells (Liu et al., 2013; Szyf, 2016). TET2 has then emerged as a key activator of gene expression (Pastor et al., 2013; Ichiyama et al., 2015). As expected, TET2 overexpression increases, but TET2 knockdown reduces, the level of 5hmC in HUVECs. Our data have shown that CSE promoter region contains a well-defined CpG island, implicating its regulation by DNA methylation and demethylation (Zhao and Han, 2009). As expected, our results showed that the methylation level of CSE promoter was decreased by TET2 overexpression and increased by the TET2 knockdown in oxLDL-treated HUVECs. Supportively, the recent studies by Li J.J. et al. (2015) and Du et al. (2016) has shown that homocysteine or oxLDL-induced DNA hypermethylation of CpG-rich region in the CSE gene promoter contributes to the decrease of the CSE/H_2_S system in macrophages.

## Conclusion

This is the first report to show that TET2 improves oxLDL-induced endothelial dysfunction through the CSE/H_2_S/NF-κB pathway. Our data also revealed that TET2 promotes DNA demethylation of the CSE gene promoter, which may be the mechanism underlying TET2 up-regulation of the CSE/H_2_S system. Our findings not only provide a new perspective on the regulation of endogenous CSE/H_2_S system but also reveal a novel role for TET2 in the protection of endothelial functions, suggesting that TET2 may become a new drug target for treating atherosclerosis.

## Author Contributions

JP, Z-HT, D-HW, L-SL, and Z-SJ conceived and designed the experiment. JP, YZ, and ZR performed the experiment and data analysis. JP, Z-HT, and BH wrote the paper. D-HW, ZW, X-LZ, and Z-SJ revised the manuscript. All authors have contributed to the final version and approved the publication of the final manuscript.

## Conflict of Interest Statement

The authors declare that the research was conducted in the absence of any commercial or financial relationships that could be construed as a potential conflict of interest.

## Supplementary Material

FIGURE S1 Effects of CSE siRNA on the CSE/H_2_S system in oxLDL-treated HUVECs with TET2 overexpression. HUVECs were transfected with or without TET2 overexpression plasmid or TET2 overexpression plasmid + CSE siRNA in the presence of oxLDL for 24 h. The expression of CSE protein **(A)** was examined via western blot analysis in cells. **(B)** The H_2_S production rates in each group of cells were determined as described in “Materials and Methods” section. **(C)** Representative fluorescent images of intracellular H_2_S level detection in each group of cells using H_2_S-specific fluorescent probes. Scale bar = 20 μm. All results are expressed as the mean ± SD of three independent experiments. ^∗^*P* < 0.05, ^∗∗^*P* < 0.01 vs. oxLDL-treated alone group. ^##^*P* < 0.01 vs. TET2 overexpression plasmid-treated group.
