# Supplementary figures and images for: TET2 Protects against oxLDL-Induced HUVEC Dysfunction by Upregulating the CSE/H2S System
Source: Front Pharmacol. 2017 Jul 26;8:486. doi: 10.3389/fphar.2017.00486 (PMC5526911; doi:10.3389/fphar.2017.00486)

Supplemental Fig. 1

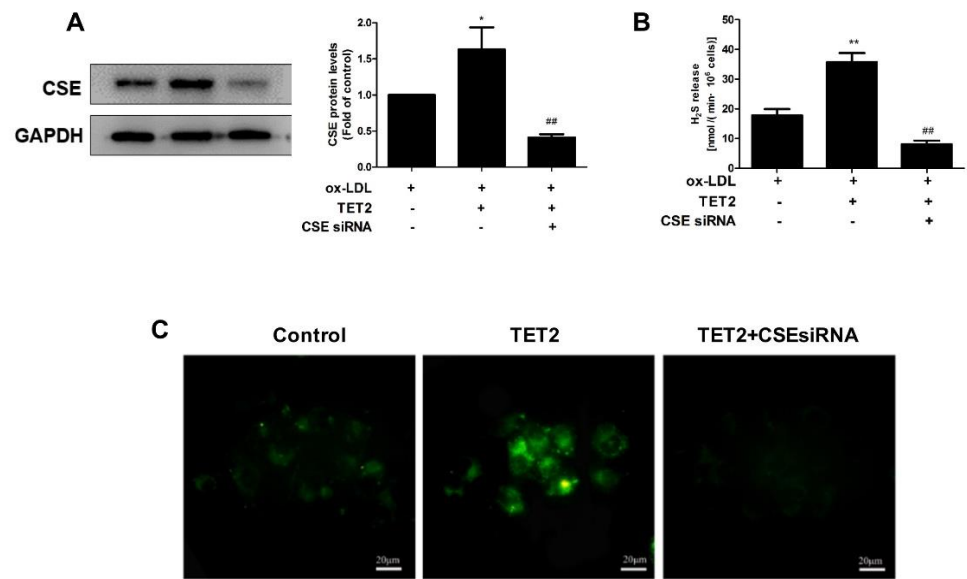

Supplement: FIGURE S1 — Effects of CSE siRNA on the CSE/H2S system in oxLDL-treated HUVECs with TET2 overexpression. HUVECs were transfected with or without TET2 overexpression plasmid or TET2 overexpression plasmid + CSE siRNA in the presence of oxLDL for 24 h. The expression of CSE protein (A) was examined via western blot analysis in cells. (B) The H2S production rates in each group of cells were determined as described in “Materials and Methods” section. (C) Representative fluorescent images of intracellular H2S level detection in each group of cells using H2S-specific fluorescent probes. Scale bar = 20 μm. All results are expressed as the mean ± SD of three independent experiments. ∗P < 0.05, ∗∗P < 0.01 vs. oxLDL-treated alone group. ##P < 0.01 vs. TET2 overexpression plasmid-treated group. [file Image_1.pdf]
