# Supplementary material for: TET2 Protects against oxLDL-Induced HUVEC Dysfunction by Upregulating the CSE/H2S System
Source: Front Pharmacol. 2017 Jul 26;8:486. doi: 10.3389/fphar.2017.00486 (PMC5526911; doi:10.3389/fphar.2017.00486)
Supplement: Supplementary file 2 [file Table_1.docx]

**Supplemental Table.1 Primer sequences used in the quantitative RT-PCR and**

**bisulfite sequencing(BSP)**

| **Gene** | **Sequences** | |
| --- | --- | --- |
| TET2 | Forward primer | 5'- ATACCCTGTATGAAGGGAAGCC -3' |
|  | Reverse primer | 5'- CTTACCCCGAAGTTACGTCTTTC -3' |
| CSE | Forward primer | 5'- GGCCTGGTGTCTGTTAATTGT -3' |
|  | Reverse primer | 5'- GCCATTCCGTTTTTGAAATGCT -3' |
| GAPDH | Forward primer | 5'- TGACTTCAACAGCGACACCCA -3' |
|  | Reverse primer | 5'- CACCCTGTTGCTGTAGCCAAA -3' |
| CSE-BSP | Forward primer | 5'-GGTTTTAATATTAAGTTAGTTTTTT -3' |
|  | Reverse primer | 5'-TTTATTAACGAATCTACAATCTCAC -3' |
